# A Deep Learning Approach for Tracking Colorectal Cancer-Derived Extracellular Vesicles in Colon and Lung Models

**DOI:** 10.1021/acsbiomaterials.5c00380

**Published:** 2025-08-26

**Authors:** Giulia Chiabotto, Bianca Dumontel, Luca Zilli, Veronica Vighetto, Giorgia Savino, Francesca Alfieri, Michela Licciardello, Massimo Cedrino, Sabrina Arena, Chiara Tonda-Turo, Gianluca Ciardelli, Valentina Cauda

**Affiliations:** † Department of Applied Science and Technology, 151697 Politecnico di Torino, Corso Duca degli Abruzzi 24, Turin 10129, Italy; ‡ Candiolo Cancer Institute, 18524 FPO-IRCCS, Candiolo, Torino 10060, Italy; § U-Care Medical s.r.l., Corso Castelfidardo 30/A, Turin 10129, Italy; ∥ POLITOBIOMed LAB, 19032 Politecnico di Torino, Turin 10129, Italy; ⊥ Molecular Biotechnology Center, 9314 University of Torino, Turin 10126, Italy; # Department of Oncology, University of Torino, Candiolo, Torino 10060, Italy; ∇ Department of Mechanical and Aerospace Engineering, Politecnico di Torino, Corso Duca degli Abruzzi 24, Turin 10129, Italy

**Keywords:** colorectal cancer, extracellular vesicles, metastasis, deep learning algorithm, 2D and 3D models

## Abstract

According to the International Agency for Research on Cancer and the World Health Organization, colorectal cancer (CRC) is the third most common cancer in the world and the main cause of gastrointestinal cancer-related deaths. Despite advances in therapeutic regimens, the incidence of metastatic CRC is increasing due to the development of resistance to conventional treatments. Metastases, particularly in the liver and lungs, represent the leading cause of death and poor prognosis in CRC patients. Recent evidence demonstrates that extracellular vesicles (EVs) are involved in communication between cancer cells and the surrounding environment. Understanding the potential mechanisms underlying EV-driven metastasis and tumor progression could facilitate the development of innovative strategies for early diagnosis and effective treatment of CRC metastasis. In this work, we developed a deep learning-based approach to track CRC-derived EVs in colon and lung models, enabling precise quantification of their uptake and trafficking *in vitro*. Moreover, we observed their tropism toward heterologous healthy cells in biologically relevant 3D models of colon and lung tissues, indicating the inherent role of CRC-EVs in metastatic niche formation and tumor initiation, raising their potential as innovative diagnostic and prognostic biomarkers as well as therapeutic targets in CRC.

## Graphical abstract

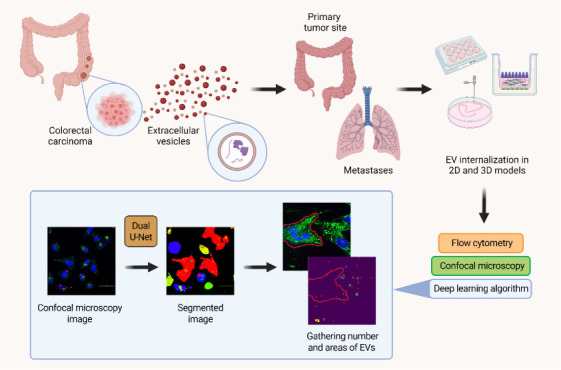

## 1 Introduction

Extracellular vesicles (EVs) are membrane-bound particles naturally secreted by cells.^1^ Acting as carriers of diverse biomolecules, including lipids, proteins, and nucleic acids, EVs mediate intercellular communication by delivering their cargo from the cell of origin to recipient cells.^2^ This process regulates numerous physiological functions and contributes to the progression of various pathological conditions, including cancer.^3^

Cancer-cell-derived EVs significantly contribute to protumorigenic processes, such as enhanced cancer cell proliferation, invasion, and extracellular matrix (ECM) remodeling.^4^ One defining feature of cancer EVs is their intrinsic “homing” capability, enabling them to preferentially interact with and be taken up by their parental cells.^5^ This preferential uptake phenomenon has been validated in various *in vitro* and *in vivo* studies. For instance, our group demonstrated that ZnO nanocrystals encapsulated in EVs from KB epidermoid carcinoma cells were preferentially internalized by the originating cancer cells.^6^ Sancho-Albero et al. showed that exosomes derived from A549 lung cancer cells, loaded with palladium, exhibited a strong preference for their progenitor cells over glioma cells.^7^ Shekh et al. demonstrated that exosomes from HCT116 colon cancer cells, when loaded with 5-fluorouracil, serve as effective drug delivery vehicles against their parental cancer cells.^8^ In a related study, Villa et al. revealed that patient-derived EVs from colorectal cancer (CRC) exhibit tumor-specific tropism, selectively targeting and inhibiting their parental tumors when loaded with therapeutic agents.^9^ Qiao et al. further confirmed the autologous tropism of exosomes from HT-1080 fibrosarcoma cells, which specifically targeted their parental cell line.^10^ Li et al. compared autologous and heterologous uptake of exosomes derived from pancreatic and lung cancer cell lines, finding significantly higher uptake efficiency in autologous conditions.^11^ Emam et al. observed that exosomes derived from murine colorectal (C26) and melanoma (B16BL6) cancer cells selectively accumulate in their respective parental cells and tumor tissues compared to exosomes from noncancerous epithelial cells.^12^

Although tumor-derived EVs exhibit a strong preference for parental cells, their targeting capability extends beyond autologous tropism. Tumor-derived EVs exhibit versatile targeting capabilities, enabling them to interact with heterologous cells.^13^ Once in circulation, EVs travel to distant tissues, where they contribute to neo-angiogenesis, ECM remodeling, and the recruitment of protumoral stromal cells, ultimately facilitating the formation of premetastatic niches.^14^ Hoshino et al. highlighted the organotropic behavior of tumor-derived EVs, demonstrating their ability to selectively deliver cargo to specific organs, such as the liver and lungs, based on integrin expression profiles.^15^ Further supporting this concept, recent findings by Cong et al. revealed that integrins α6 and β4 on CRC-derived EVs play a pivotal role in promoting lung metastasis by facilitating niche formation in the lung microenvironment.^16^ Given that CRC is one of the leading causes of cancer-related mortality worldwide,^17^ understanding how CRC-derived EVs drive metastasis colonization is essential to improve therapeutic strategies and patient prognosis.^18^

Three-dimensional (3D) culture models have recently emerged as valuable tools to investigate the interactions between cells and EVs *in vitro*.^19,20^ Unlike traditional two-dimensional (2D) cultures, 3D models provide a more physiologically relevant environment that closely mimics the structural and functional complexity of human tissues.^21^ This makes 3D culture models ideal for studying EV biodistribution and their impact on cell behavior and tissue architecture, offering deeper insights into their role in cancer progression. Furthermore, these 3D models allow direct optical fluorescence imaging and immune staining, enabling the possible monitoring of EV biodistribution and effects in a time-step manner and under different scenarios.

Always in the field of biomedical imaging, deep learning approaches—a subset of artificial intelligence—have transformed image analysis by offering robust tools to extract complex, high-dimensional patterns from large data sets^22^ across various imaging modalities, including computed tomography (CT), magnetic resonance imaging (MRI), and digital optical microscopy. These models excel in tasks, such as image segmentation, classification, and feature extraction,^23,24^ underscoring their versatility in diverse biomedical applications. Beyond Convolutional Neural Networks (CNNs), recent advancements have explored other effective “end-to-end” deep learning techniques, particularly the novel transformer architecture.^25,26^ While this approach delivers superior performance across various tasks, it demands extensive high-quality labeled medical data for optimal training and fine-tuning.

To address the well-known scarcity of high-quality annotated data, particularly in the medical and biomedical fields, multiple CNN-based neural networks have been utilized in complex segmentation and detection systems.^27,28^ These models offer a comprehensive understanding of the spatial context within images, significantly enhancing their performance in complex biomedical applications and tissue imaging. Derived from the U-Net framework,^29^ these techniques have demonstrated outstanding efficacy in segmenting small cells, even in experimental and noise-prone environments.

In this context, deep learning’s ability to automate image segmentation and analysis with exceptional precision not only minimizes human error but also enhances reproducibility, making it a crucial tool for exploring the complex dynamics of EVs in cancer metastasis.

In this scenario, we leverage the current knowledge about tumor-derived EVs with the use of a deep learning algorithm for automated image analysis and tracking, and we also explore EV trafficking in 3D models of CRC and lung. Thus, the aim of this work is to analyze the localization of EVs, isolated from human CRC cells (CRC-EVs), first in 2D models of healthy and tumor colon and lung cell lines. Then, a deep learning algorithm is employed in the 2D setup to track EV internalization amount and rate in target cells. These data are not only validated with conventional biological techniques, like flow cytometry, but also provide insights into EVs’ role in metastasis. We then approach a more complex scenario by adopting 3D models of colon and lung tissues as proof of the previously proven concepts. Here, we explore the EV biodistribution in tridimensional volumes and observe how CRC-EVs migrate and target different cells, mainly healthy ones, indicating a net tendency for their metastatic potential.

## 2 Materials and Methods

### 2.1 Cell Culture

The human colon fibroblast cell line CCD-18Co (CRL-1459) and the human lung fibroblast cell line MRC-5 (CCL-171) were cultured in StableCell MEM (Sigma-Aldrich St. Louis, MO, United States) supplemented with 10% fetal bovine serum (FBS, Gibco, Thermo Fisher Scientific, Carlsbad, CA, United States), 1% MEM amino acid solution (Sigma-Aldrich), and 1% sodium pyruvate (Sigma-Aldrich). Human colon carcinoma cells Colo-320DM (CCL-220) were cultured in RPMI 1640 (Sigma-Aldrich) containing 10% FBS. Human lung epithelial cells A549 (CCL-185) were cultured in DMEM high glucose (Sigma-Aldrich) supplemented with 10% FBS. All cells were purchased from ATCC and grown as adherent monolayers in T75 flasks (Corning, VWR International, Milano, Italy) in the presence of 1% penicillin/streptomycin (Sigma-Aldrich) and 1% l-glutamine 200 mM (Lonza) at 37 °C under a 5% CO2 atm.

### 2.2 Generation of the 3D Model of Colon

#### 2.2.1 Preparation of Bioink

The bioink preparation and the creation of the 3D model of colon were carried out according to our recent publication.^200^ To prepare the bioink, gelatin from porcine skin (type-A, 300 bloom) and alginic acid sodium salt from brown algae (medium-viscosity) were dissolved in CCD-18Co culture medium (StableCell MEM supplemented with 10% FBS) to make 11% (w/v) and 2.5% (w/v) solutions, respectively. The solutions were stirred at 50 °C until homogeneity was reached. For the cross-linking, solutions of 10% (w/v) microbial transglutaminase, purchased from Ajiomoto North America, Inc. (Fort Lee, NJ, USA), and 4% (w/v) CaCl_2_ were prepared in culture medium and filtered using the Stericup Quick Release-HV Sterile Vacuum Filtration system (Corning). All solutions were stored at 4 °C until use.

The bioprinting system comprised two microfluidic syringe pumps (Model DUAL-NE-1000, KF Technology), loaded with two syringes containing the hydrogel precursor and a 2% CaCl_2_ solution. These syringes were connected to the external and internal channels of a commercial coaxial needle with 17G and 23G nozzles, respectively. The extrusion speeds were set to 100 μL/min for the bioink and 200 μL/min for the CaCl_2_ solution. The entire process was performed under sterile conditions. Before bioprinting, all equipment was cleaned with 70% ethanol and subjected to UV radiation for 30 min for sterilization. The coaxial needle was sterilized by rinsing it with sodium hypochlorite, PBS, and 99% ethanol solution. To maintain the bioink at its optimal viscosity for printing, a heater was used to keep the temperature around 28–30 °C.

#### 2.2.2 Healthy Colon 3D Model

To obtain cell-laden tubular conduits with cells embedded in the bioink, CCD-18Co cells were resuspended in the bioink polymer, which had been previously heated to 45 °C and centrifuged at 500 *g* for 2 min to remove any bubbles, at a concentration of 2 × 10^6^ cells/mL. The bioink and a 2% CaCl_2_ solution were transferred into 10 and 20 mL syringes, respectively, and connected to the outer and central channel inlets of the coaxial nozzle using silicone tubing. Once placed on separate syringe pumps, the bioink and the 2% CaCl_2_ solution were extruded simultaneously. The bioprinted tubes were collected in a 150 × 15 mm Petri dish filled with 1% CaCl_2_ solution. At the end of this process, the bath solution was replaced with a 2% transglutaminase solution to further cross-link the conduits overnight, prior to incubation at 37 °C in 5% CO_2_.

#### 2.2.3 Colorectal Cancer 3D Model

Human colorectal cancer cells, Colo-320DM, were resuspended at a concentration of 1 × 10^7^ cells/mL in RPMI 1640 cell culture medium and seeded into hollow tubes cut into segments of 5–6 cm in length, 2 days after extrusion. The Colo-320DM suspension was loaded into a 1 mL syringe and injected into the lumen of the bioprinted tubes using a sterile hypodermic 26G needle. After cell seeding, the bioprinted conduits were placed in a 24-well plate, with 1 mL of a mixed medium prepared by combining StableCell MEM and RPMI 1640 in a 1:1 ratio, which was changed every 2 days. The coculture was incubated at 37 °C in an atmosphere of 5% CO_2_ for 4 days until the cells formed a compact structure.

### 2.3 Generation of the 3D Model of Lung

Biomimetic electrospun membranes were prepared as previously described.^30^ Briefly, a blended solution (total polymer concentration of 15% wt./v) was prepared by dispersing polycaprolactone (PCL) (*M_n_* = 80 kDa, Sigma-Aldrich) and gelatin Type A (Sigma-Aldrich) in a weight ratio of 80:20 wt./wt. in a mixture of acetic acid (Fisher Scientific) and formic acid (Sigma-Aldrich) (50:50 v/v), as described.^31^ The solution was loaded into a glass syringe, and the electrospinning process was performed using the NovaSpider instrument (CIC nanoGUNE, San Sebastián, Spain) employing previously optimized parameters: 12 kV voltage, 500 μL/h flow rate, and 12 cm needle-to-collector distance.

The developed electrospun membranes (coded as PCL-Gel) were integrated onto the commercial 12-well inserts, as previously described.^30^ Briefly, each transwell insert (SABEU GmbH and Co. KG) was modified by removing the commercial polyethylene terephthalate (PET) membrane and leaving only the insert support. Then, PCL-Gel membrane specimens were attached to the external wall of each transwell insert through a thin layer of polydimethylsiloxane (PDMS; Sylgard 184, VWR).

Collagen hydrogel was prepared following the manufacturer’s instructions. Briefly, the prehydrogel solution was obtained by mixing type I bovine collagen solution (10 mg/mL, FibriCol, Advanced Biomatrix, Carlsbad) with 10× PBS and sterile distilled water. The acidic pH of the solution was adjusted to 7.5 by adding 0.1 M NaOH at 4 °C. MRC-5 fibroblasts were embedded into the collagen prehydrogel solution at 1.5 × 10^6^ cells/mL. Then, 180 μL of the solution was poured into the apical compartment of the PCL-Gel transwell and incubated at 37 °C for 30 min to promote the sol–gel transition. Then, A549 cells were suspended in 0.3 mL of EMEM medium at 1.4 × 10^5^ cells/cm^2^ and seeded atop the collagen hydrogel. After 3 days, the medium in the apical chamber was removed, and the cell coculture was maintained at the air–liquid interface (ALI) for an additional 7 days, replacing the medium every 2 days in the basolateral chamber.

### 2.4 Extracellular Vesicle Isolation and Characterization

To collect EVs, Colo-320DM cells were seeded in cell culture dishes (Corning) at a density of 10,000 cells/cm^2^ and cultured under standard conditions until they reached 80% confluence. Then, the medium was replaced with RPMI medium supplemented with 10% EV-depleted FBS, prepared by overnight ultracentrifugation as previously described.^32^ The following day, the cell supernatant was collected and processed through a series of differential centrifugation steps at 4 °C, based on the protocol by Théry et al.^32^ Briefly, the conditioned medium was first centrifuged at 300 *g* for 10 min to remove cells. The resulting supernatant was then centrifuged at 2,000 *g* for 20 min to eliminate cell debris, followed by centrifugation at 10,000 *g* for 30 min to remove apoptotic bodies and large vesicles. The clarified supernatant was transferred to polypropylene tubes (29.9 mL Optiseal tubes, Beckman Coulter) and ultracentrifuged at 100,000 *g* for 70 min using an Optima Max-XP Ultracentrifuge with an MLA50 rotor (Beckman Coulter, Fullerton, CA, USA). The pellet was resuspended in sterile, chilled 0.1 μm-filtered phosphate-buffered saline (PBS), centrifuged again at 100,000 *g* for 60 min, and resuspended in sterile, chilled 0.1 μm-filtered physiological saline (0.9% NaCl, NovaSelect) supplemented with 1% dimethyl sulfoxide (DMSO, Sigma-Aldrich). The final EV preparation was stored at −80 °C until further use in subsequent experiments.

EV concentration and size were assessed by using the NanoSight NS300 instrument (NanoSight Ltd., Amesbury, UK), equipped with a 505 nm laser beam and a NanoSight syringe pump. For each EV preparation, three 60 s videos were recorded with an infusion rate of 50 au and a camera level value between 14 and 16 were analyzed using the Nanoparticle Tracking Analysis Software (NTA version 3.4). Hydrodynamic size distribution and z-potential were measured using the Dynamic Light Scattering (DLS) technique with a Zetasizer Nano ZS90 (Malvern Instruments) equipped with a 633 nm He–Ne laser. EVs (50 μL) were diluted in 950 μL of physiological solution and analyzed in triplicate at 25 °C.

The protein concentration of the isolated EVs was determined using the Bradford assay, as previously described.^32^ Bradford reagent (Bio-Rad, Hercules, CA, USA) was diluted 1:5 in deionized water and added to EV samples, which were first diluted 1:2 in 0.1 μm-filtered PBS. Serially diluted bovine serum albumin (BSA, Sigma-Aldrich) standards with known concentrations were prepared alongside the samples. Absorbance was measured at 590 nm using a microplate spectrophotometer (Multiskan GO, Thermo Fisher Scientific), and the protein concentration of the EV samples was calculated by comparing their absorbance values with the calibration curve generated from the BSA standards. All measurements were performed in triplicate.

The EVs’ morphology was analyzed through Transmission Electron Microscopy (TEM) using a Talos F200X G2 (Thermo Fisher Scientific) instrument at an operating voltage of 80 kV. EV samples were spotted onto Formvar/carbon 200-mesh nickel grids (Electron Microscopy Sciences, Hatfield, PA, USA) and left to adhere for 20 min. Samples were then fixed with a solution of 2.5% glutaraldehyde, washed with PBS and sterile H_2_O, and stained with Nano-W and NanoVan (Nanoprobes, Yaphank, NY, USA), as previously reported.^33^

To evaluate the presence of exosomal and nonexosomal proteins, EVs and parental Colo320DM cells were lysed on ice using RIPA buffer supplemented with protease and phosphatase inhibitors (Sigma-Aldrich). Protein extracts (10 μg per sample) were separated by electrophoresis on 4–12% gradient NuPAGE precast gels (Invitrogen, Carlsbad, CA, USA) under reducing conditions and transferred onto 0.2 μm nitrocellulose membranes using the Transblot Turbo Transfer System (Bio-Rad). Membranes were blocked with 5% nonfat milk (Sigma-Aldrich) for 2 h at room temperature, followed by overnight incubation at 4 °C with the following primary antibodies: mouse anti-Alix and mouse anti-β-actin (both from Santa Cruz Biotechnology, Dallas, TX, USA), rabbit anti-HSP90α (Abcam, Cambridge, UK), and rabbit anti-GM130 (Cell Signaling Technology, Danvers, MA, USA). Details of all antibodies are listed in Table S1. After extensive washing in 0.1% Tween-20 in TBS (TBS-T), membranes were incubated with horseradish peroxidase-conjugated secondary antibodies (Jackson ImmunoResearch Laboratories, Inc., West Grove, PA, USA) for 1 h. Protein bands were detected by using the Clarity Max Western ECL Substrate and visualized with the Chemidoc imaging system (Bio-Rad).

Furthermore, the expression of tetraspanins CD63 and CD81 by EVs was characterized by cytofluorimetric analysis. As previously described,^32,34^ approximately 5 μg (5 × 10^9^ particles) of EVs were coupled with 10 μL of Aldehyde/Sulfate Latex Beads, 4% w/v, 3 μm (Thermo Fisher Scientific), for 2 h on a test tube rotator at RT in a total volume of 1 mL of PBS. Then, 110 μL of glycine solution (1 M in PBS, Sigma-Aldrich) were added to saturate the remaining binding sites, and samples were washed three times and redispersed in PBS + 0.5% BSA. EVs immobilized on beads were then incubated with the PE-conjugated antihuman CD63 antibody or the APC-conjugated antihuman CD81 antibody, and the corresponding isotype controls (all purchased from BioLegend and listed in Table S2) for 30 min at 4 °C, protected from light. After two washing steps, samples were analyzed using a Guava easyCyte 6–2L flow cytometer (Millipore, Merck). A total of 5,000 events from singlet-gated beads were recorded per sample at a low flow rate (0.12 μL/s). Representative graphs were generated with FCS Express, and results are expressed as Median Fluorescence Intensity (MFI) normalized by background subtraction of the respective isotype control.

### 2.5 Cytotoxicity Assay

Cytotoxicity was evaluated in healthy and tumor cell lines treated with varying concentrations of EVs. CCD-18Co, MRC-5, Colo-320DM, and A549 were seeded (2 × 10^3^, 1 × 10^4^, 5 × 10^3^, and 1.25 × 10^3^, respectively) into 96-well flat-bottom culture plates (Greiner Bio-One) and exposed to EVs at concentrations of 10, 20, and 50 μg/mL for 24, 48, and 72 h. At 22, 46, and 70 h of incubation, 10 μL of WST-1 reagent (CELLPRORO Roche) was added to each well, and after an additional 2 h, formazan absorbance was measured at 450 nm using a microplate spectrophotometer, with a reference wavelength of 620 nm. All experiments were conducted in triplicate for each cell line, and the results were normalized against untreated controls.

### 2.6 Cellular Internalization of EVs

Flow cytometry was used to assess the internalization of EVs by the treated cells. EVs were labeled with the fluorescent probe 1,1-dioctadecyl-3,3,3,3-tetramethylindodicarbocyanine (DiD, Thermo Fisher Scientific) at a concentration of 5 μg/mL and washed using Amicon Ultra-0.5 Centrifugal Filters (50 kDa MWCO, Millipore, Merck). Following centrifugation, the EVs were resuspended in 100 μL of cell culture medium and incubated with cells (at a number of cells per well equal to 1.2 × 10^4^ for CCD-18Co, 6 × 10^4^ for MRC-5, 3 × 10^4^ for Colo-320DM, and 3 × 10^4^ for A549) in 24-well flat-bottom culture plates (Greiner Bio-One). After 5, 24, and 48 h of incubation, the cells were collected, washed with PBS, and resuspended in 250 μL of PBS for flow cytometric analysis. At least 10^4^ events were recorded using a Guava flow cytometer at a flow rate of 0.59 μL/s, excluding debris. Analysis was performed with a red laser (λ_ex_ = 642 nm) detecting fluorescence intensity shifts in the Red-R channel (emission filter 661/15). The results were analyzed using Guava InCyte Software (Millipore, Merck) and expressed in terms of mean fluorescence intensity (MFI) and percentage of positive events compared with untreated controls. For comparison, the internalization of EVs derived from healthy fibroblasts and synthetic liposomes was also analyzed. EVs were isolated from CCD-18Co cells following the same protocol described for CRC-EVs. Synthetic liposomes were prepared by the extrusion method using a mini-extruder (Avanti Polar Lipids) with a 100 nm polycarbonate membrane. Briefly, the lipid mixture (molar ratio: 50% DOPA/10% DOPC/38.5% Cholesterol/1.5% DSPE-PEG(2000)-Amine/0.15% DiD) was dried under vacuum and then rehydrated with physiological solution at a final concentration of 1 mg/mL to allow the formation of liposomes. The suspension was shaken (200 rpm) at 40 °C for 45 min and subsequently extruded through the membrane 13 times to obtain uniformly sized liposomes.

For fluorescence microscopy analysis, 3 × 10^4^ cells were seeded in 4-well chamber slides (Thermo Fisher Scientific, Nunc Lab-Tek II CC2 Chamber Slide System) and treated with CRC-EVs labeled with DiD (10 μg/mL) as described above. After 24 h, cell membranes were stained by incubating the cells with Alexa Fluor 488-conjugated Wheat Germ Agglutinin (WGA488, λ_ex_ = 495 nm, Thermo Fisher Scientific) diluted in cell medium at a concentration of 4 μg/mL for 10 min at 37 °C. Following PBS washes, nuclei were stained with Hoechst 33342 (Thermo Fisher Scientific) diluted in cell medium at a concentration of 2.5 μg/mL for 5 min at 37 °C. The cells were washed twice with PBS before immediate analysis. Images were captured using a spinning disk fluorescence-inverted microscope (Eclipse TiE, Nikon) with a 60× immersion oil objective.

Timelapse experiments were performed using the same instrument coupled with a stage-top chamber connected to temperature and CO_2_ controllers (Okolab). Cell membranes and nuclei were labeled as previously described, immediately prior to the administration of DiD-labeled EVs. Images were collected at selected time steps, starting from 30 min after EVs administration and continuing up to 4 h. Images were acquired in all the fluorescence channels used, every 5 min for the first hour and every 10 min for the next 3 h, and were used as input for the algorithm.

### 2.7 Cell and EV Segmentation System

As a tailored approach, tested on both lung and colon cells’ interaction with EVs, the original Dual U-Net^35^ architecture was implemented with a modified loss and postprocessing phase for optimal particle delineation. To enhance training efficacy, priority was given to balancing cells and EVs while manually annotating only the highest-quality elements from each image. Building upon the optimal results obtained from the dual-branch architecture, the original distance transformation prediction for the regressive branch and categorical cell borders for the classification branch output were maintained. A smooth L1 loss function in the regressive branch was used to maintain numerical stability and mitigate scale-dependent inconsistencies. Analyzing the initial training results, challenges in the dual-branch architecture concerning both the categorical and regressive loss functions were observed, specifically the weighted cross-entropy and L1 losses. In response, data set characteristics were systematically adjusted—ranging from single-channel illumination to the number of annotated elements—until no further performance gains were observed. To improve segmentation accuracy, the Dice loss function^36^ was introduced in the categorical branches to address variations in cell and EV dimensions and volumes. Meanwhile, a smooth L1 loss function was employed in the regressive branch to enhance numerical stability and mitigate inconsistencies related to scale differences. During the training preprocessing phase, common data augmentation strategies were employed, namely flipping, contrast modification, scaling, and the introduction of blur and noise, with application probabilities set between 0.25 and 0.3.

The pipeline incorporates a single trained U-Net-based neural network, efficiently trained on an aggregated channel encompassing all particles. At test time, this network independently infers segmentation for cells, EVs, and cell nuclei, which are subsequently merged to optimize EV counting per cell. The final phase, called ″fusion postprocessing” is crucial to mitigate segmentation artifacts, particularly addressing challenges posed by sparse cell borders, which were especially problematic in cell segmentation, despite the generalizability of the U-Net approach.

### 2.8 EV Uptake in 3D Models

Healthy and CRC colon 3D models were transferred into 4-well chamber slides (Thermo Fisher Scientific Nunc Lab-Tek II CC2 Chamber Slide System) and treated with 3.6 × 10^9^ particles/well of DiD-labeled EVs, prepared as described earlier, for 24 h. After EV incubation, the 3D models were fixed for 30 min using Image-iT Fixative Solution (4% formaldehyde, methanol-free, Thermo Fisher Scientific) and then washed with PBS. Cell membranes were stained with Alexa Fluor 488-conjugated Wheat Germ Agglutinin (WGA488, λ_ex_ = 495 nm, Thermo Fisher Scientific) diluted in PBS containing 0.1% BSA (Sigma-Aldrich) at a concentration of 5 μg/mL for 1 h at room temperature. After thorough PBS washes, nuclei were stained with Hoechst (Thermo Fisher Scientific) at a concentration of 1 μg/mL in PBS for 30 min at room temperature. The samples were then washed twice with PBS and imaged using a spinning disk fluorescence-inverted microscope (Eclipse TiE, Nikon) with 4× and 20× objectives.

After 6 days of culture at ALI, the lung 3D model was treated with fluorescently labeled EVs prepared as described earlier. A total of 1.75 × 10^9^ particles/well were administered in the lower chamber of the well, and the plate was incubated for 24 h on an orbital shaker (50 rpm). Then, the PCL-Gel transwell insert was rinsed with PBS and fixed for 30 min. Following PBS washes, 0.1% v/v Triton X-100 in PBS was applied to both the apical and basolateral compartments for 10 min to permeabilize the cells. The sample was then blocked with 1% v/v BSA in PBS for 1 h, followed by incubation with Alexa Fluor 488-conjugated Phalloidin (1:400 dilution in 1% v/v BSA) for 40 min at room temperature in the dark. DAPI (1:1000 in PBS) was then added to both compartments for nuclear staining, followed by a 5 min incubation. After staining, the PCL-Gel membranes were carefully detached from the inserts and mounted on glass coverslips using Fluoromount Aqueous Mounting Medium. Images were acquired using a spinning disk fluorescence Eclipse Ti2 inverted microscope (Nikon) with a 60× immersion oil objective.

## 3 Results and Discussion

### 3.1 EV Isolation and Characterization

EVs were isolated from Colo-320DM cells using a sterile differential ultracentrifugation protocol^32^ and characterized according to MISEV guidelines.^37^ The size and morphology were assessed by NTA, DLS, and TEM analyses on EVs collected from multiple isolation rounds. TEM images revealed a population of round-shaped vesicles with the characteristic cup-shaped morphology of stained EVs and an average size of approximately 100 nm (Figure 1A). NTA further confirmed the size distribution of EVs in physiological solution, identifying a main peak centered at 130 nm, as representatively shown in Figure 1B, and measuring an average particle concentration of 3.74 × 10^10^ ± 8.48 × 10^9^ particles/mL (mean ± S.E.). As expected, the number size distribution obtained by DLS (Figure 1C) corresponded to NTA measurements, with a single peak centered at 138.7 nm. In contrast, the *Z*-average and intensity size distribution showed values over 300 nm, accounting for the presence of larger particles or aggregates which, although fewer in number, heavily contribute to the scattering signal due to the strong dependence of scattering intensity on particle size. Indeed, the PdI value, equal to 0.277, indicated moderate polydispersity of the sample, suggesting partial aggregation or size heterogenicity, typical of complex biological samples such as EVs. The z-potential was also analyzed (Figure 1D), registering a negative surface charge of −9.49 mV, in line with literature results.

**Figure 1:**
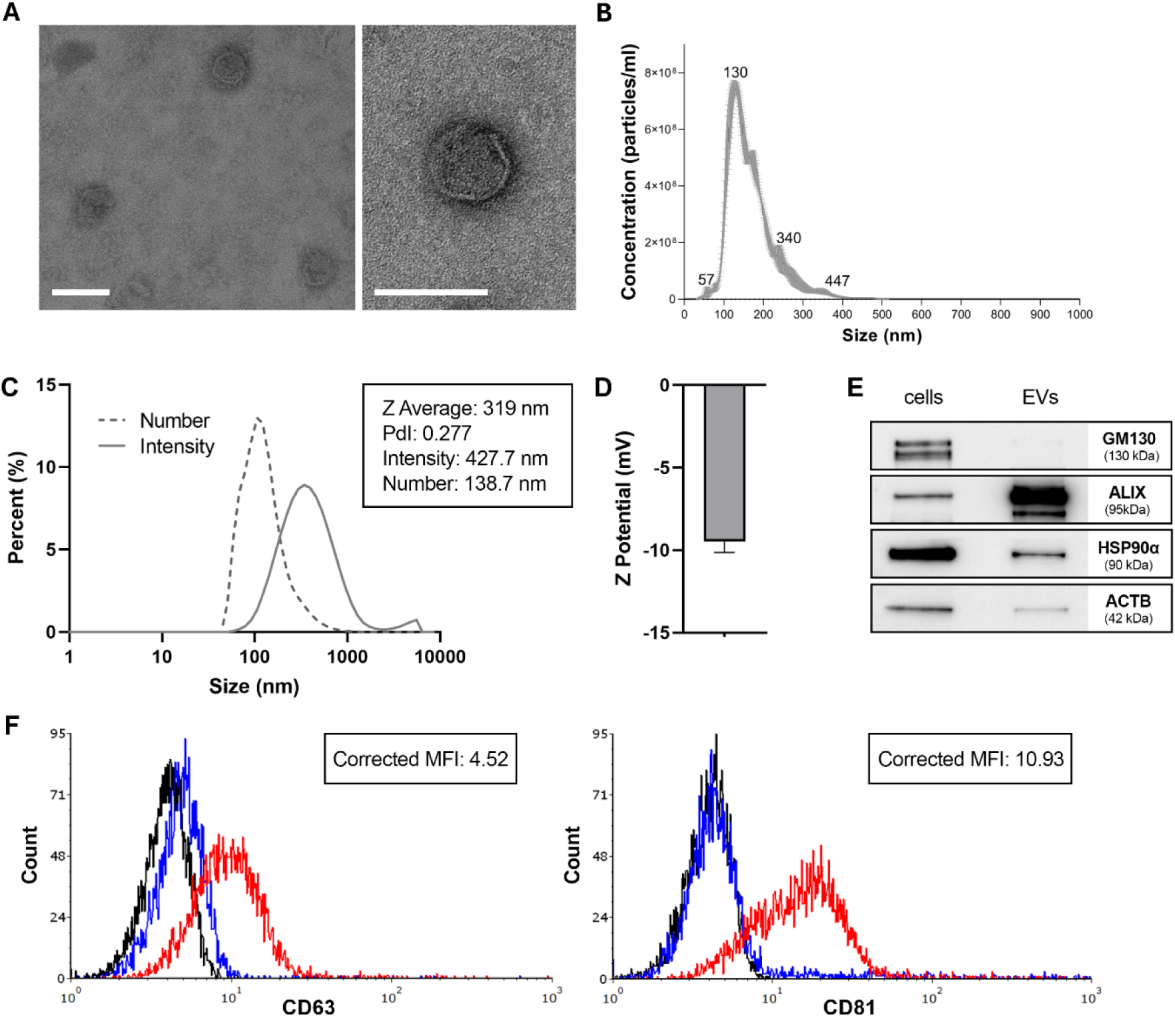
Characterization of CRC-EVs. **A)** Representative TEM micrographs of EVs negatively stained with Nano-W and Nano-Van (scale bar: 100 nm). **B)** NTA measurement of EVs dispersed in physiological solution, showing size distribution. **C)** DLS number and intensity distribution of EVs in physiological solution. **D)** Z-potential analysis demonstrated a surface charge of −9.49 mV. **E)** Western blot analysis of CRC-EVs and their parental cells confirmed the expression of cytosolic proteins ALIX, HSP90α, and ACTB, and the absence of the Golgi marker GM130. **F)** Flow cytometry analysis of classical exosomal markers CD63 and CD81 expressed on EVs isolated from Colo-320DM. Black lines represent unstained EV-beads, blue lines represent EV-beads incubated with isotype control antibodies, and red lines represent EV-beads incubated with CD63-PE or CD81-APC antibodies. Corrected MFI values were calculated by subtracting the MFI of the isotype control from the MFI of the specific antibody. Data are representative of more than 10 independent EV isolation batches.

Using the Bradford assay, a protein concentration of 122 ± 15 μg/mL (mean ± S.E.) was assessed in 10 independent EV batches. Western blot analysis confirmed the presence of canonical EV-associated cytosolic proteins ALIX, HSP90α, and ACTB in the isolated EVs, while the Golgi marker GM130 was undetectable, excluding contamination by cellular components and confirming the purity of the EV preparations (Figure 1E). Additionally, the expression of the typical exosomal markers CD63 and CD81 was evaluated in EVs by flow cytometry analysis (Figure 1F). These results confirm that the isolation protocol yielded a highly uniform EV population, in terms of both size and morphology, consistent with established characteristics of exosomes. The observed particle size and marker expression align well with previously reported data for EVs from CRC cells, further validating the reproducibility and reliability of the isolation and characterization methods used.

### 3.2 EV Cytotoxicity and Targeting in Healthy and Tumor Cells

The interaction between CRC-EVs and their parental cell line (Colo-320DM) and healthy colon cells (CCD18-Co) was assessed. Additionally, to evaluate their potential role in promoting metastasis, CRC-EVs were incubated with healthy lung fibroblasts (MRC-5) and a lung cancer cell line (A549), commonly used as an *in vitro* model of alveolar epithelium.^38,39^

Before internalization experiments, CRC-EVs were incubated at varying doses (10, 20, and 50 μg/mL) with four cell lines at three different time points (24, 48, and 72 h) to assess potential cytotoxic effects. As summarized in Figure S1, cell viability showed no statistically significant differences across all tested conditions over the treatment period, indicating minimal cytotoxic effects of CRC-EVs within the tested range. These data align with previous reports highlighting the functional role of EVs as modulators of the tumor microenvironment rather than direct mediators of cell death.^5^ Based on these findings, an intermediate dose of 20 μg/mL was selected for subsequent experiments.

The internalization of CRC-EVs was assessed by using flow cytometry analysis (Figure 2A,B) and fluorescence microscopy (Figure 2C,D). A high level of uptake was observed in all four cell lines at the three treatment time points (5, 24, and 48 h), with nearly 100% EV-positive cells for all tested conditions and only a slightly lower extent of internalization for healthy colon cells at 5 h (Figure 2A). However, a comparison of the median fluorescence intensity (MFI) revealed cell-specific differences in the uptake efficiency of CRC-EVs. Significantly higher MFI values, which correlated with a higher amount of internalized EVs, were observed in healthy lung fibroblasts (MRC-5) after 5 h and in healthy colon cells (CCD18-Co) after longer incubation times (Figure 2B).

**Figure 2:**
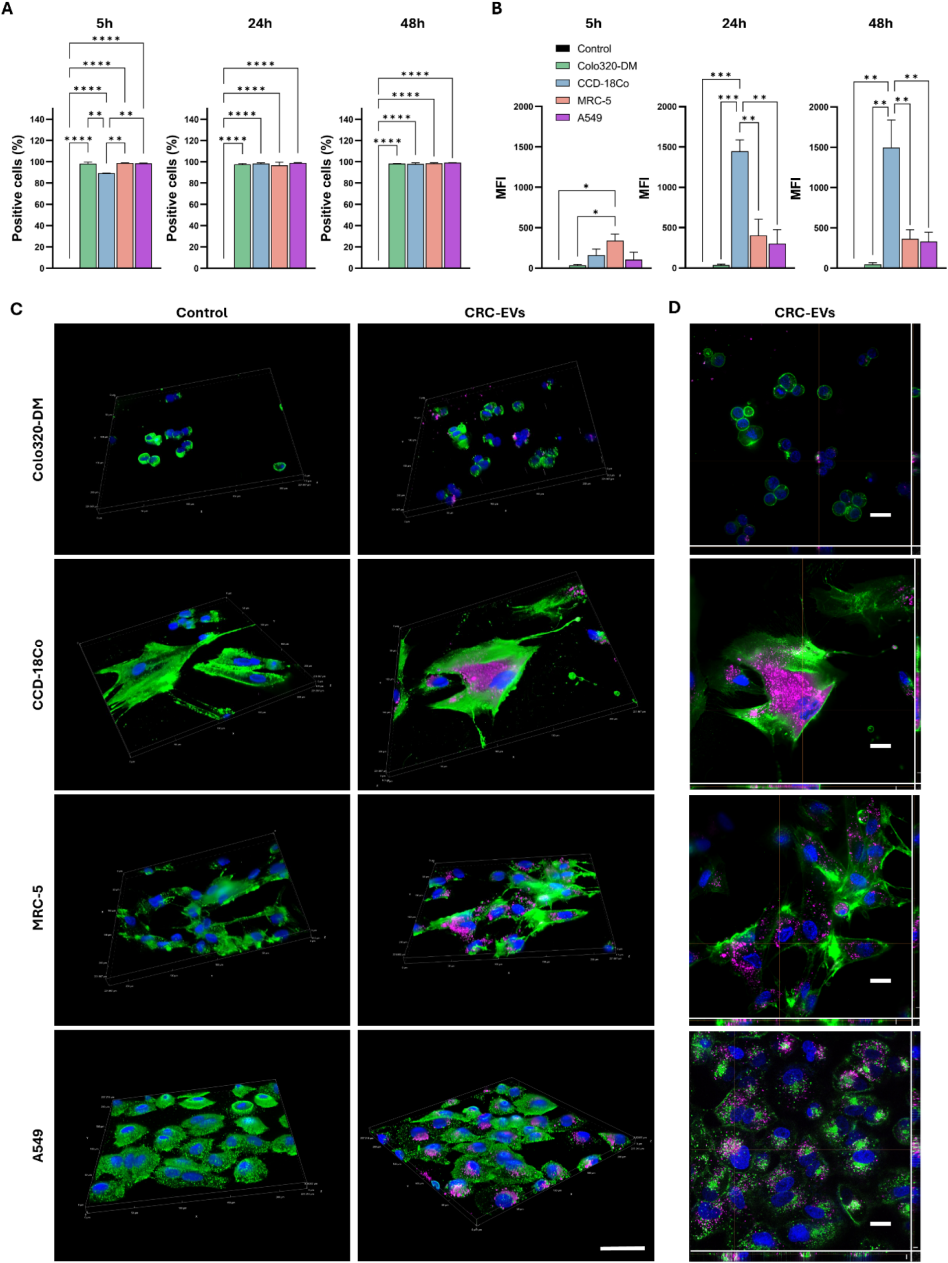
Internalization of CRC-EVs in colon and lung cells. **A)** Percentage of EV-positive cells in colon (Colo-320DM and CCD-18Co) and lung (A549 and MRC-5) cell lines treated with 20 μg/mL of CRC-EVs for 5, 24, and 48 h, as determined by flow cytometry. **B)** Median fluorescence intensity (MFI) of internalized CRC-EVs in the four cell lines highlights cell-specific differences in uptake efficiency. Data are presented as mean ± SE from two independent experiments. Statistical significance: *p < 0.05, **p < 0.01, ***p < 0.001, ****p < 0.0001 using one-way ANOVA. **C)** 3D reconstructions from z-stack images and **D)** representative z-stack slices of CRC-EV internalization in colon and lung cells at 24 h. Scale bar: 50 μm for images in panel C and 20 μm for images in panel D.

To further assess the specificity of CRC-EV internalization for healthy colon cells, we compared the uptake of EVs derived from Colo320DM cells and CCD-18Co fibroblasts in normal and tumor colon cell lines (Figure S2). CRC-EVs exhibited selective internalization into CCD-18Co fibroblasts while showing minimal uptake in the parental Colo320DM tumor cells, indicating a pronounced tropism toward the healthy fibroblast population. In contrast, EVs isolated from CCD-18Co were internalized by both cell types, with a preference for reentry into the fibroblast line from which they originated. As a negative control, synthetic liposomes were prepared with a negative z-potential similar to that of the EVs. They were efficiently internalized by all tested cell lines without displaying any marked cell-type specificity. Together, these results support the selective targeting properties of the CRC-derived EVs.

These results were further corroborated by fluorescence microscopy: 3D-reconstructed images from z-stack projections (Figure 2C) acquired 24 h after EV administration and representative 3D slices (Figure 2D) clearly showed CRC-EVs internalized in the perinuclear region of all four cell types. The highest EV internalization was observed in the healthy colon cell line CCD-18Co, whereas the lowest uptake was recorded in the parental Colo-320DM cell line. The pronounced uptake in CCD-18Co cells may reflect their role as healthy colon fibroblasts, potentially providing a supportive microenvironment for protumoral changes initiated by EVs. Notably, EV internalization in both lung-derived healthy fibroblasts (MRC-5) and alveolar epithelial (A549) cells was higher than that measured in CRC parental cells, suggesting a potential lung tropism of CRC-EVs. These results align with the hypothesis of CRC-EVs exhibiting organotropic behavior, particularly toward lung tissues, to establish metastatic niches, and are consistent with previous evidence linking EV tropism to integrin expression profiles on their surface.^15,16^

### 3.3 EV Tracking by Deep Learning-Based Approach

The interaction between CRC-EVs and colon and lung cells was further analyzed in time-lapse by fluorescence microscopy experiments, tracking the uptake from 30 min postadministration up to 4 h. Representative images of the four cell lines during EV internalization are shown in Figures S3 and S4.

In this setup, a deep learning algorithm was then employed to track the amount and rate of internalization of CRC-EVs in target cells. The developed method allows segmentation and quantification of EVs within the delineated cells across the 2D image data sets, enabling a comprehensive analysis of EV behavior during interactions with different cell types. It combines deep learning frameworks with a supplementary pipeline that gathers EV counts and calculates their coverage as a percentage of the total cell area, leveraging the segmentation results from the primary algorithm, as schematically depicted in Figure 3.

**Figure 3:**
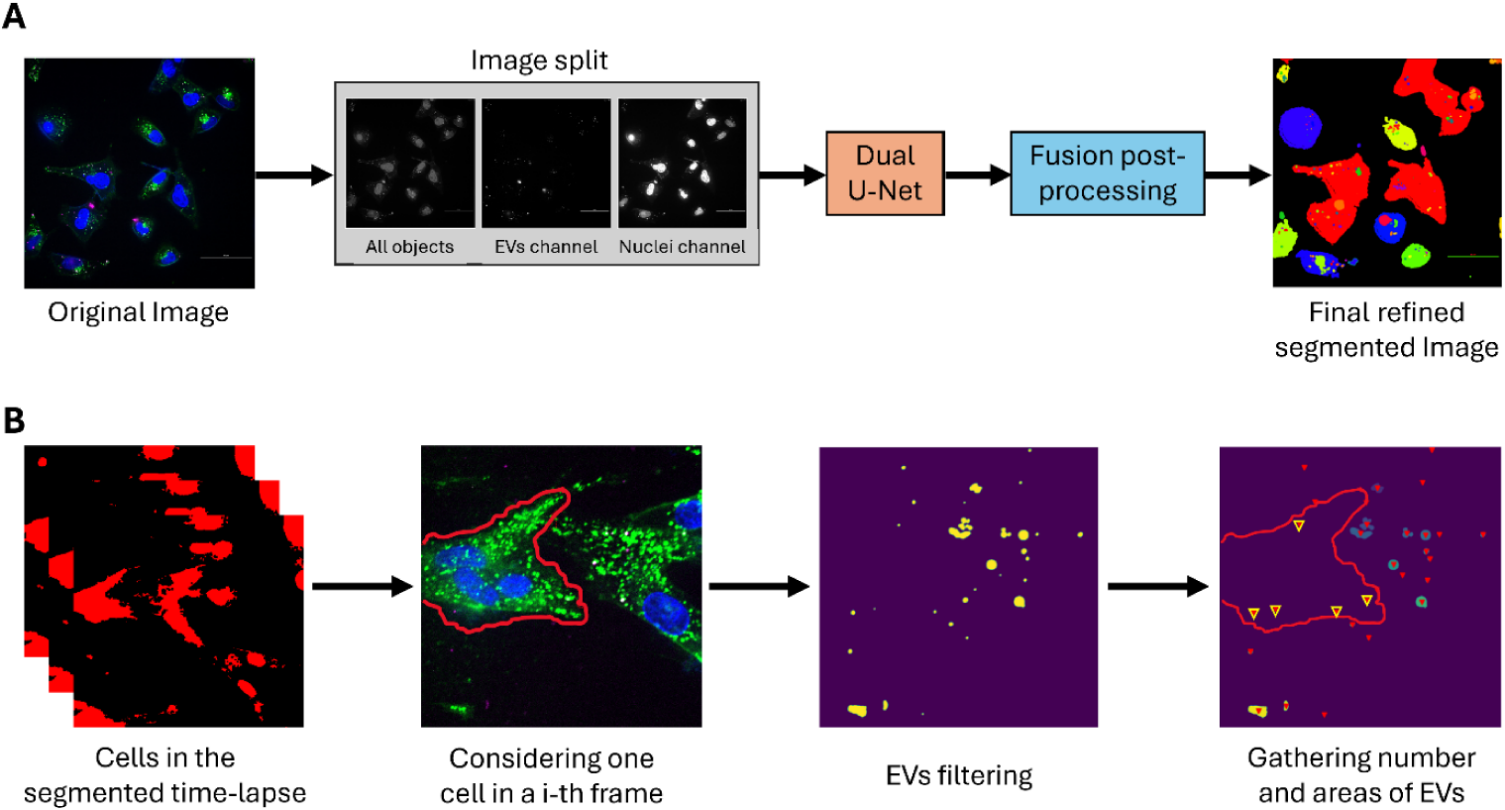
Cell and EV segmentation using AI. **A**) Segmentation pipeline starting with the initial fluorescence microscopy image at a specific time point, which is divided into three distinct single channels (nuclei, EVs, and merge channel). Following a triple-prediction process, the resulting outputs are combined to yield the final refined results. **B**) EVs counting pipeline: after collecting the segmented cell images in the time-lapse series, the single cell is delineated (see the red line) at each time step and for every cell image. Then, EVs are recognized, and the segmented EVs are further separated into internal and external components with respect to the cell body (red line in the last image, with internalized EVs depicted as yellow triangles). Then, the internalized EVs are counted for each segmented cell for every image and every time frame.

The pipeline for cell and EV segmentation from 2D fluorescence microscopy images draws inspiration from original U-Net-based architectures.^35,40,41^ The studies explored underscored the critical role of data set quality and how CNN pipelines integrate multiple neural networks with postprocessing phases to achieve accurate cell segmentation and detection.^42,43^ This combination has proven to be effective in handling diverse image settings, yielding promising results. As described in Figure 3A, our deep learning framework incorporates the Dual U-Net architecture and employs a triple-prediction strategy during inference, utilizing three distinct input channels derived from the RGB images in the data set of fluorescence optical images collected at different time steps. To refine segmentation accuracy, a final fusion-based postprocessing step is applied, effectively addressing the overlap between cell nuclei and CRC-EVs. The developed postprocessing phase is built around a single tunable hyperparameter, allowing for straightforward fine-tuning to enhance particle delineation accuracy. This design ensures a well-balanced trade-off between automation and accuracy, effectively adapting to changes in image exposure and contrast under different experimental settings.

Across all experiments, a fixed subset of cells was chosen for each segmented time-lapse to gather key metrics for monitoring EV internalization over time (Figure 3B). The primary metric consists of a straightforward count of segmented EVs within the selected cell masks (segmented areas depicted with a red line). To better address the challenges posed by clustered EVs, we also measured the total EV area within the segmented cells for each frame. By combining these two metrics, our approach offers a more precise analysis, especially in the initial phase of the experiments, considering the difficult segmentation conditions.

Images from the four data sets corresponding to the four cell lines tested were used to evaluate the performance of the computational model before applying the counting pipeline. Six images from each data set were used for the training and validation of the improvement in the loss function, while three images from each data set formed the segmentation test set. In this phase, separate inferences on the segmentation of cells and EVs were performed, as detailed in the Materials and Methods section, and the number of annotated cells and vesicles varied between different experiments, with an average of 5 elements for cells or cell clusters and around 5 or 10 EVs in the experiments with colon or lung cells, respectively.

The results obtained are listed in Figure 4. The count of segmented EVs was divided by the number of nuclei present in the segmented cell area to obtain a normalized value of the EV count/cell (Figure 4A). This shows a higher accumulation of CRC-EVs in lung cells (first in MRC-5 healthy fibroblasts and second in A549 cells, as a model of alveolar epithelium) compared to healthy colon cells. Especially, in CRC parental cells, almost no internalized EVs were counted. As well, the percentages of the total area covered by EVs within the segmented cells (Figure 4B) show similar trends, with negligible values for healthy and tumoral colon cells and higher values for lung cells. Both metrics indicated a higher internalization rate for healthy lung fibroblasts (MRC-5). This result is fairly aligned with what was already observed by flow cytometry analysis at 5 h, where the MRC-5 cell line had the highest MFI value (Figure 2B). These data similarities show that the developed algorithm was able to provide reliable results, in good accordance with conventional techniques.

**Figure 4:**
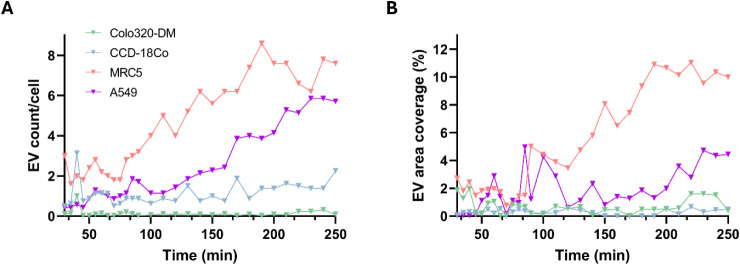
EV tracking in time-lapse imaging of colon and lung cells. **A)** Number of internalized CRC-EVs and **B)** percentages of EV area coverage within the segmented cells determined by the developed deep learning algorithm from timelapse frames monitoring the uptake from 30 min after administration up to 4 h in the four different cell lines.

### 3.4 EV Uptake in 3D Models of Healthy and Tumor Tissues

To further validate our findings in a more physiologically relevant context, we employed 3D *in vitro* models of colon and lung tissues, which provide a higher level of complexity compared to traditional 2D cultures.

We developed a biomimetic 3D model of healthy colon tissue by bioprinting a colon-like tubular structure. The bioink consisted of gelatin and alginate, in which CCD-18Co fibroblasts were suspended. To achieve controlled cross-linking, the bioink was mixed with transglutaminase and CaCl_2_, then extruded using a coaxial needle, forming a self-supporting structure that mimics the architecture of the native colon (Figure 5A). To replicate tumor formation within the colon, we subsequently introduced Colo-320DM cells, mimicking the progression from a healthy to malignant state (Figure 5B).

**Figure 5:**
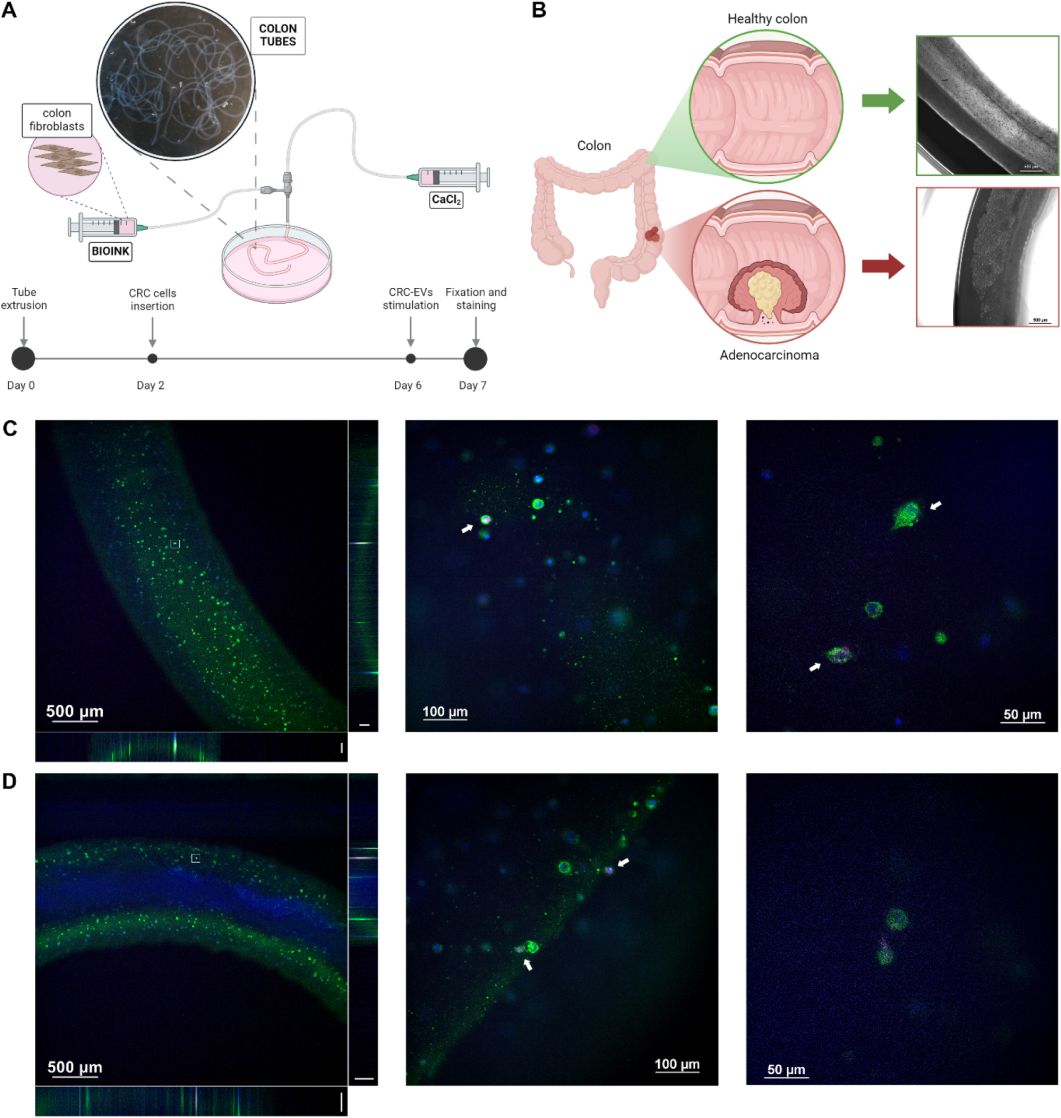
Internalization of CRC-EVs in 3D models of healthy colon and CRC. **A**) Schematic representation of the 3D colon model generation, illustrating the bioprinting process and the timeline of the experimental setup. **B**) Illustration of healthy and CRC colon models, with bright-field images (magnification 4×) showing the 3D structure of the bioprinted healthy colon (top, green border) and CRC colon (bottom, red border). **C,D**) Confocal microscopy imaging of EV internalization in the healthy colon model (C) and CRC colon model (D). Z-stack projection (magnification 4×, lateral projections scale bar: 100 μm) provides an overview of EV distribution (left panel in C and D), while higher magnification images (20× central panel and 40× right panel) show EV uptake at the cellular level. Illustrations are created using BioRender.

After 24 h of incubation, fluorescence imaging revealed that CRC-EVs effectively infiltrated the 3D healthy colon structures (Figures 5C and S5A,B). Similarly, in the 3D CRC model, EV internalization was predominantly observed at the outer borders of the colon tubes, where CCD-18Co fibroblasts reside, while minimal uptake was detected within the tubes, where CRC cells are located (Figures 5D and S5C,D). The preference for EV internalization by healthy fibroblasts over CRC cells is consistent with our previous observations in 2D cultures, further supporting the hypothesis that CRC-EVs interact with nonmalignant cells, potentially priming them to support tumor progression.

To investigate the metastatic potential of CRC-EVs, we established a 3D lung model using a biomimetic transwell-like system that supports ALI culture conditions^30^ (Figure 6A). This system closely mimics the lung alveolus, incorporating a bioartificial PCL-Gel electrospun membrane in place of commercial transwell membranes to provide a physiologically relevant microenvironment. As previously reported,^30^ ALI culture conditions can induce the expression of alveolar epithelial markers and the secretion of mucus by A549 cells.

**Figure 6:**
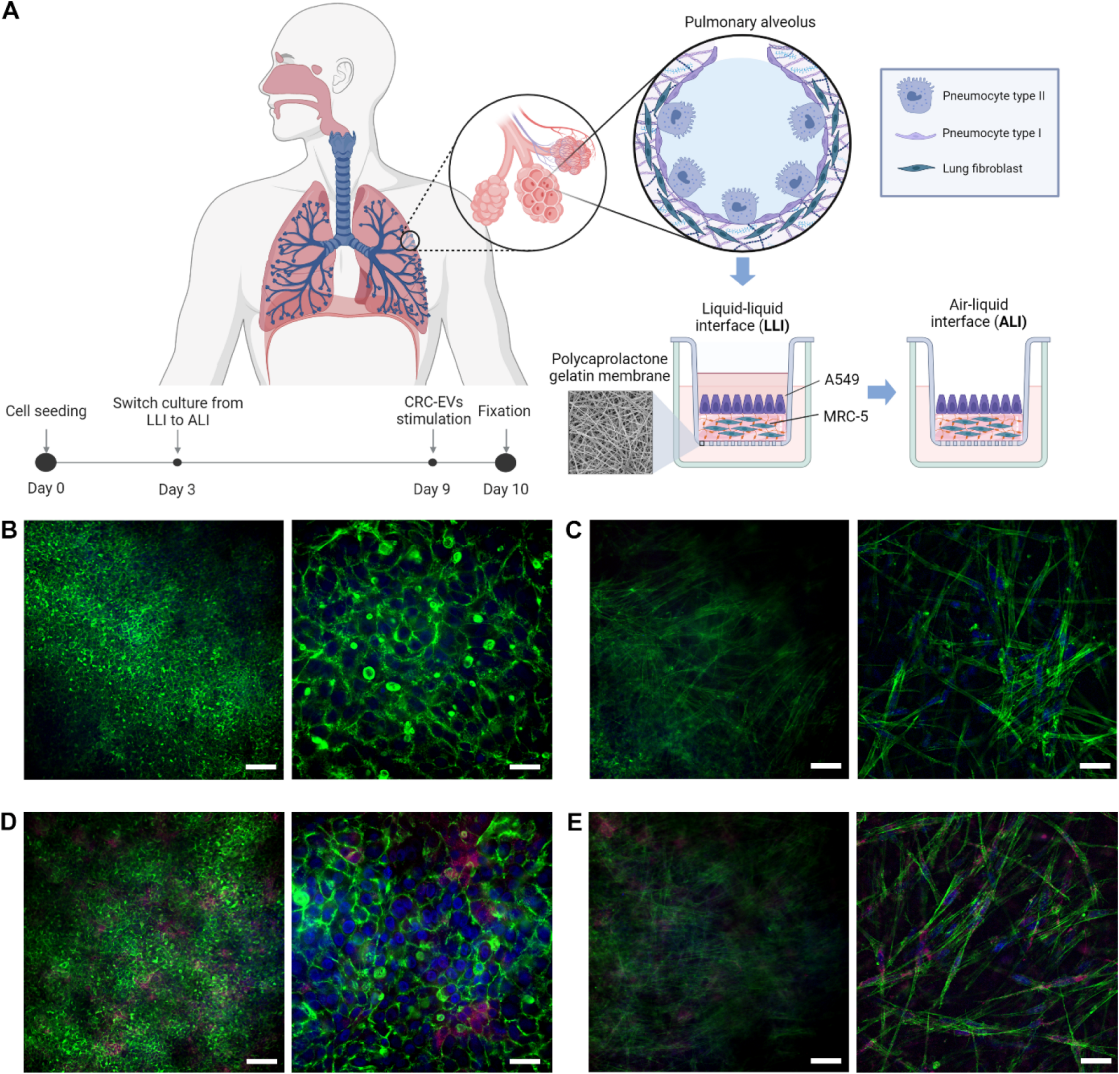
Internalization of CRC-EVs in a 3D model of lung. **A**) Schematic representation of the 3D lung model generation, showing the biomimetic transwell-like system with air–liquid interface (ALI) culture conditions, and the timeline of the experimental setup created using BioRender. **B,C**) Confocal microscopy images of A549 epithelial cells (B) and MRC-5 fibroblasts within the hydrogel (C). **D,E**) Confocal microscopy images of A549 epithelial cells (D) and MRC-5 fibroblasts (E) after 24 h of incubation with CRC-EVs. Magnification: left panels 20× (scale bar 100 μm) and right panels 60× (scale bar 20 μm).

The coculture model was established by seeding MRC-5 fibroblasts within a collagen hydrogel on the apical side of the PCL-Gel membrane, followed by the addition of A549 cells atop the MRC-5-laden hydrogel. The ALI was introduced 3 days after seeding and maintained for an additional 6 days before EV administration. Confocal microscopy images confirmed the presence of both A549 (Figure 6B) and MRC-5 (Figure 6C) cells within the hydrogel. Fluorescence staining of the cytoskeleton highlighted the distinct morphology of each cell type: under ALI conditions, A549 cells formed a uniform layer with a polygonal epithelial-like morphology, while MRC-5 fibroblasts retained their characteristic elongated, spindle-like shape. Additionally, the video reconstruction (Video S1) from z-stack image overlays further validated the coexistence of A549 cells and MRC-5 fibroblasts within the 3D coculture system.

To mimic the physiological route of EV dissemination via circulation, CRC-EVs were administered from the basolateral side of the transwell. After 24 h of incubation, CRC-EVs exhibited efficient penetration into the tissue structure, with consistent accumulation both in epithelial cells (Figures 6D and S6A,B) and in fibroblasts (Figures 6E and S6C,D). Moreover, the video reconstruction (Video S2) from z-stack image overlays further confirmed the internalization of CRC-EVs in A549 cells and MRC-5 fibroblasts within the 3D coculture system. These findings further support our 2D culture results and strengthen the role of CRC-EVs in premetastatic niche formation by actively interacting with noncancerous cells in distant organs.

## 4 Conclusions

In this study, we demonstrate the ability of CRC-EVs to interact with colon and lung cells and infiltrate complex 3D tissues, thereby supporting their role in metastatic processes. The preferential internalization of CRC-EVs by nonmalignant cells, particularly fibroblasts, underscores their functional role in tumor progression and premetastatic niche formation. Our findings further emphasize the critical role of CRC-EVs in potentially conditioning the surrounding microenvironment to facilitate metastasis.

Furthermore, our deep learning-based approach for tracking EVs *in vitro* offers valuable insights into EV trafficking in CRC. By monitoring EV–cell interactions, our algorithm enables a more comprehensive understanding of the spatiotemporal distribution of CRC-EVs and their impact on tumor microenvironment modulation.

The investigation focused on stromal fibroblasts, which are key mediators of tumor progression and metastasis. Nonetheless, the incorporation of normal epithelial and endothelial cells in future studies will help clarify the specificity of EV targeting and uptake mechanisms across different cell types and enhance the translational relevance of our findings.

Finally, our study highlights the importance of integrating advanced imaging and computational tools to investigate EV behavior *in vitro*, paving the way for novel diagnostic, prognostic, and therapeutic strategies targeting EV-mediated communication within the tumor microenvironment.

## Supplementary Material







## Notes

Z-stack image overlays further validated the coexistence of A549 cells and MRC-5 fibroblasts within the 3D coculture system.(MP4)
Z-stack image overlays further confirmed the internalization of CRC-EVs in A549 cells and MRC-5 fibroblasts within the 3D coculture system.(MP4)
Table S1 and S2 reporting the list of antibodies used for western blot analysis and flow cytometry. Figure S1 showing cytotoxicity of CRC-EVs in healthy and tumor cells; Figure S2 showing the comparison between the uptake of CRC-EVs, EVs from healthy fibroblasts, and synthetic liposomes in healthy and tumor cells; Figures S3 and S4 showing fluorescence microscopy images of EV interaction with colon and lung cells at different time steps; Figures S5 and S6 showing fluorescence microscopy images of EV uptake by healthy and CRC colon 3D models and by the lung 3D model.(PDF)
