# Supplementary material for: A Deep Learning Approach for Tracking Colorectal Cancer-Derived Extracellular Vesicles in Colon and Lung Models
Source: ACS Biomater Sci Eng. 2025 Aug 26;11(9):5343–55. doi: 10.1021/acsbiomaterials.5c00380 (PMC12421507; doi:10.1021/acsbiomaterials.5c00380)
Supplement: Supplementary file 3 [file ab5c00380_si_003.pdf]

# Supporting Information

# A deep learning approach for tracking colorectal cancer-derived extracellular vesicles in colon and lung models

*Giulia Chiabotto[1,2]‡, Bianca Dumontel[1]‡, Luca Zilli[3], Veronica Vighetto[1], Giorgia Savino[1], Francesca Alfieri[3], Michela Licciardello[4], Massimo Cedrino[5], Sabrina Arena[2,6], Chiara Tonda-Turo[4,7], Gianluca Ciardelli[4,7], Valentina Cauda[1*]*

[1]Department of Applied Science and Technology, Politecnico di Torino, Corso Duca degli Abruzzi 24, 10129 Turin, Italy.

[2]Candiolo Cancer Institute, FPO-IRCCS, 10060 Candiolo, TO, Italy.

[3]U-Care Medical s.r.l., Corso Castelfidardo 30/A, 10129 Turin, Italy.

[4]POLITOBIOMed LAB, Politecnico di Torino, Turin, Italy

[5]Molecular Biotechnology Center, University of Torino, 10126 Turin, Italy.

[6]Department of Oncology, University of Torino, 10060 Candiolo, TO, Italy.

[7]Department of Mechanical and Aerospace Engineering, Politecnico di Torino, Corso Duca degli Abruzzi 24, 10129 Turin, Italy.

‡ Equally contributing authors

* Corresponding author: Prof. Valentina Cauda, phone: +390110907389, e-mail: valentina.cauda@polito.it

Table S1. List of antibodies used for western blot analysis

| Target Protein | Species | Supplier | Catalogue Number | Dilution | Washing Buffer |
|---|---|---|---|---|---|
| Alix | Mouse | Santa Cruz Biotechnology | sc-271975 | 1:200 | TBS-T |
| Beta-actin | Mouse | Santa Cruz Biotechnology | sc-47778 | 1:1000 | TBS-T |
| HSP90α | Rabbit | Abcam | ab2928 | 1:1000 | TBS-T |
| GM130 | Rabbit | Cell Signaling Technology | #12480 | 1:1000 | TBS-T |
| Anti-mouse IgG-HRP | Goat | Jackson ImmunoResearch | 115-035-003 | 1:10000 | TBS-T |
| Anti-rabbit IgG-HRP | Goat | Jackson ImmunoResearch | 111-035-003 | 1:10000 | TBS-T |

Table S2. List of antibodies used for flow cytometry analysis

| Target Protein | Species | Supplier | Catalogue Number | Conjugate | Dilution | Washing Buffer |
|---|---|---|---|---|---|---|
| CD63 | Mouse | BioLegend | 353004 | PE | 1:10 | PBS+ 0,5% BSA |
| CD81 | Mouse | BioLegend | 349510 | APC | 1:20 | PBS+ 0,5% BSA |
| Mouse IgG1, κ (Isotype ctrl, PE) | Mouse | BioLegend | 400114 | PE | 1:20 | PBS+ 0,5% BSA |
| Mouse IgG1, κ (Isotype ctrl, APC) | Mouse | BioLegend | 400122 | APC | 1:20 | PBS+ 0,5% BSA |

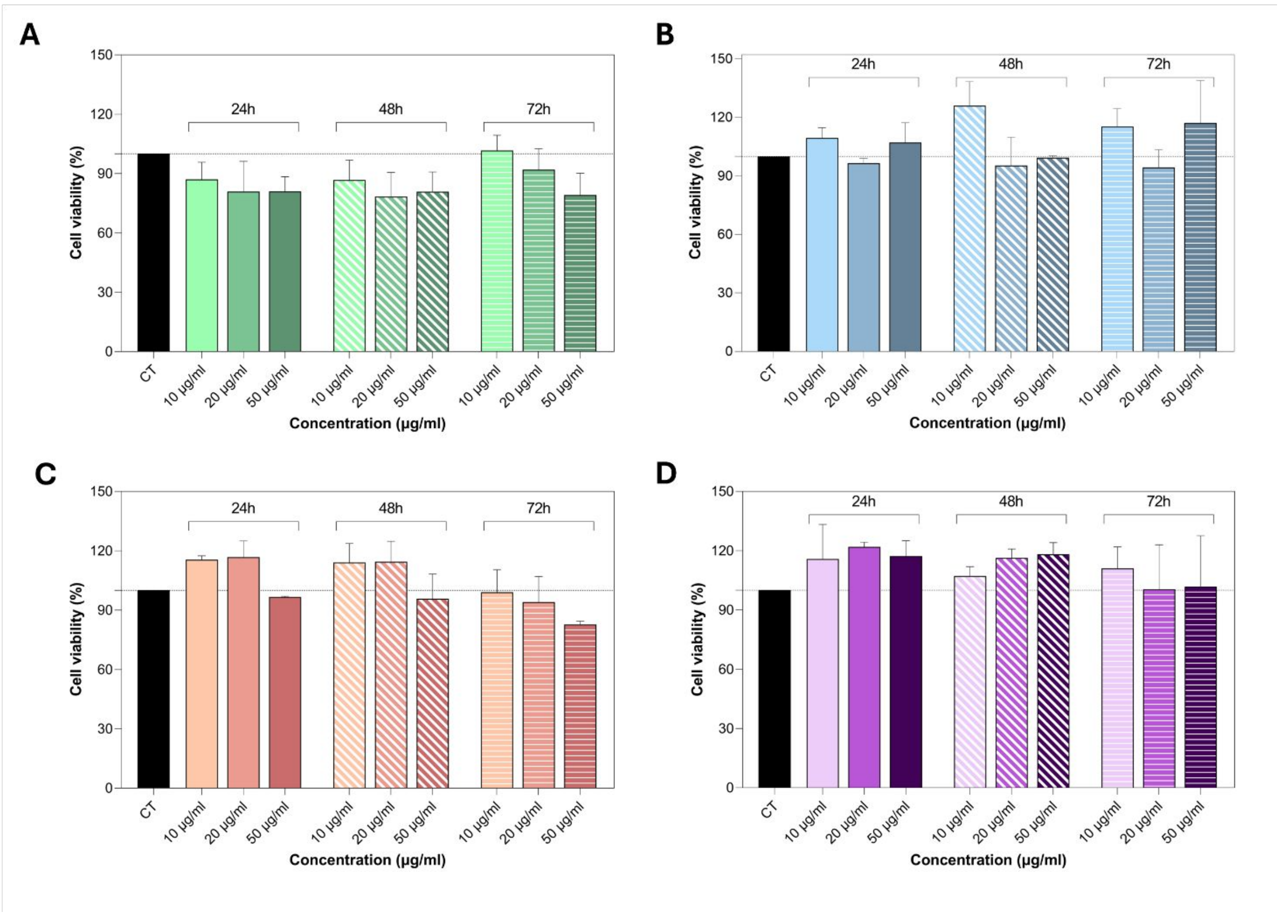


**Figure S1. Cytotoxicity of CRC-EVs in healthy and tumor cells.** Cytotoxicity assay of CRC-EVs at varying doses (10, 20, and 50 µg/mL) over three time points (24, 48, and 72 hours) in **A)** parental CRC cells (Colo-320DM), **B)** healthy colon fibroblasts (CCD-18Co), **C)** healthy lung fibroblasts (MRC-5) and **D)** lung cancer cells (A549), as an in vitro model of alveolar epithelium. Cell viability was expressed as a percentage relative to untreated controls. Data are presented as mean ± S.E. from two independent experiments. Statistical analysis was performed using two-way ANOVA, determining no significant differences.

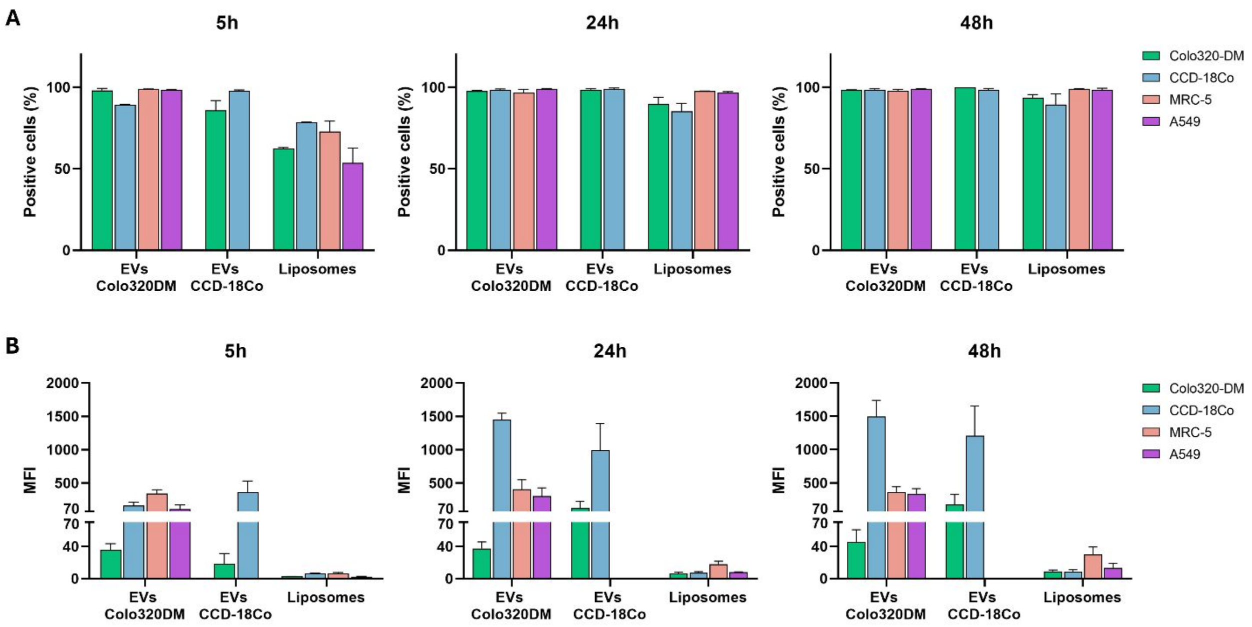


**Figure S2. Uptake of EVs derived from CRC cells (Colo320DM) and healthy fibroblasts (CCD-18Co) or of synthetic liposomes in healthy and tumor cells. A)** Percentage of positive events and **B)** Median fluorescence intensity (MFI) of cells treated with 20 μg/mL of EVs derived from Colo320DM and CCD-18Co cells or equivalent amount of synthetic liposomes for 5, 24, and 48 hours as determined by flow cytometry. Synthetic liposomes with surface charge and size (-14.2 mV and 134.3 nm as measured by Zeta Potential and NTA technique, respectively), comparable to those of natural EVs, were included as a negative control and administered at 1 μg/mL to achieve a concentration in part/mL similar to that of EVs. Data are presented as mean ± S.E. from at least two independent experiments

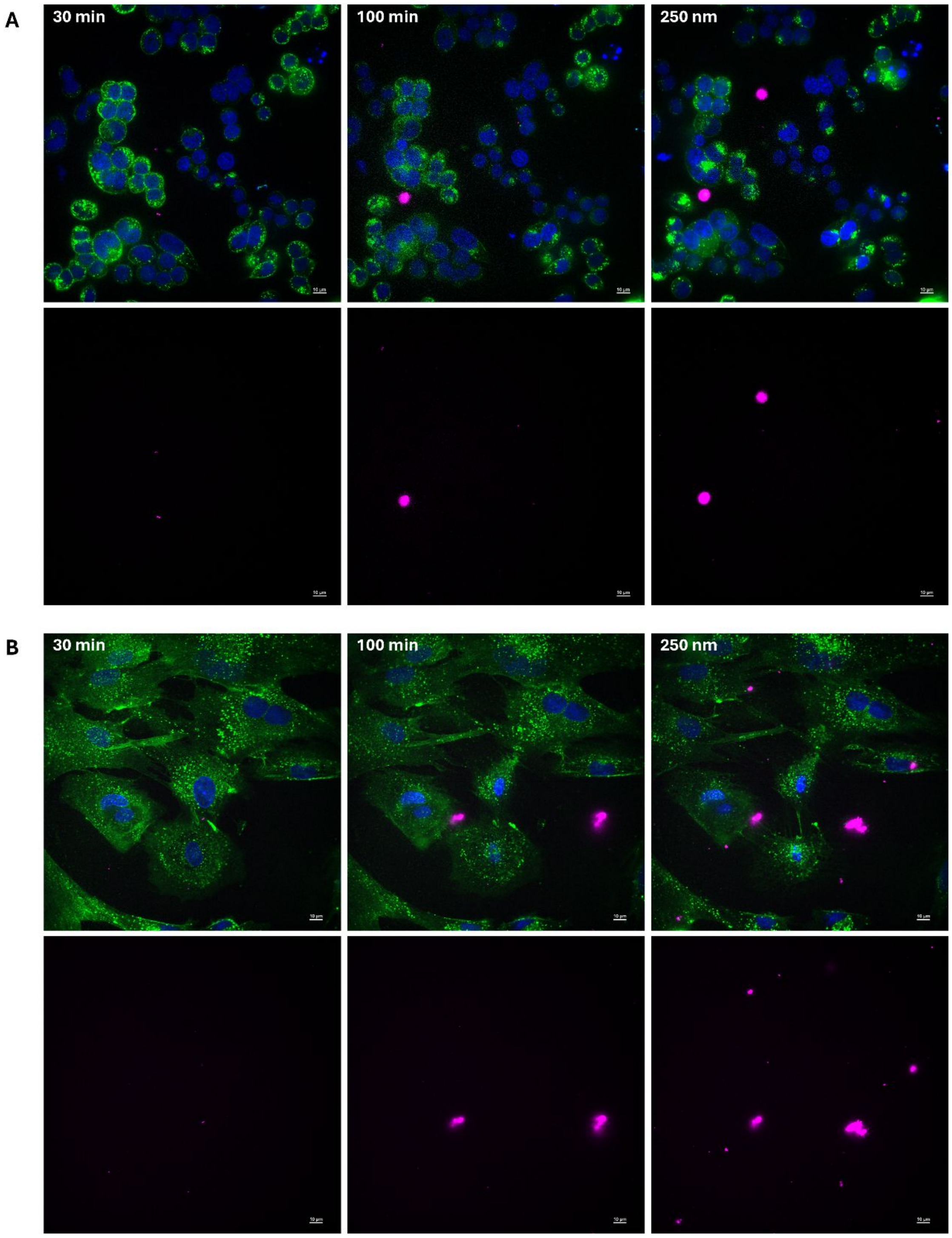


**Figure S3. Images at different time points of fluorescence microscopy time-lapse of CRC-EVs uptaken by colon cells.** Merged and EV channel images from the first (30 min), intermediate (100

min) and last (250 min) frames of time lapse experiments monitoring CRC-EV internalization in **A)** Colo-320DM and **B)** CCD-18Co cells. Scale bars: 10µm.

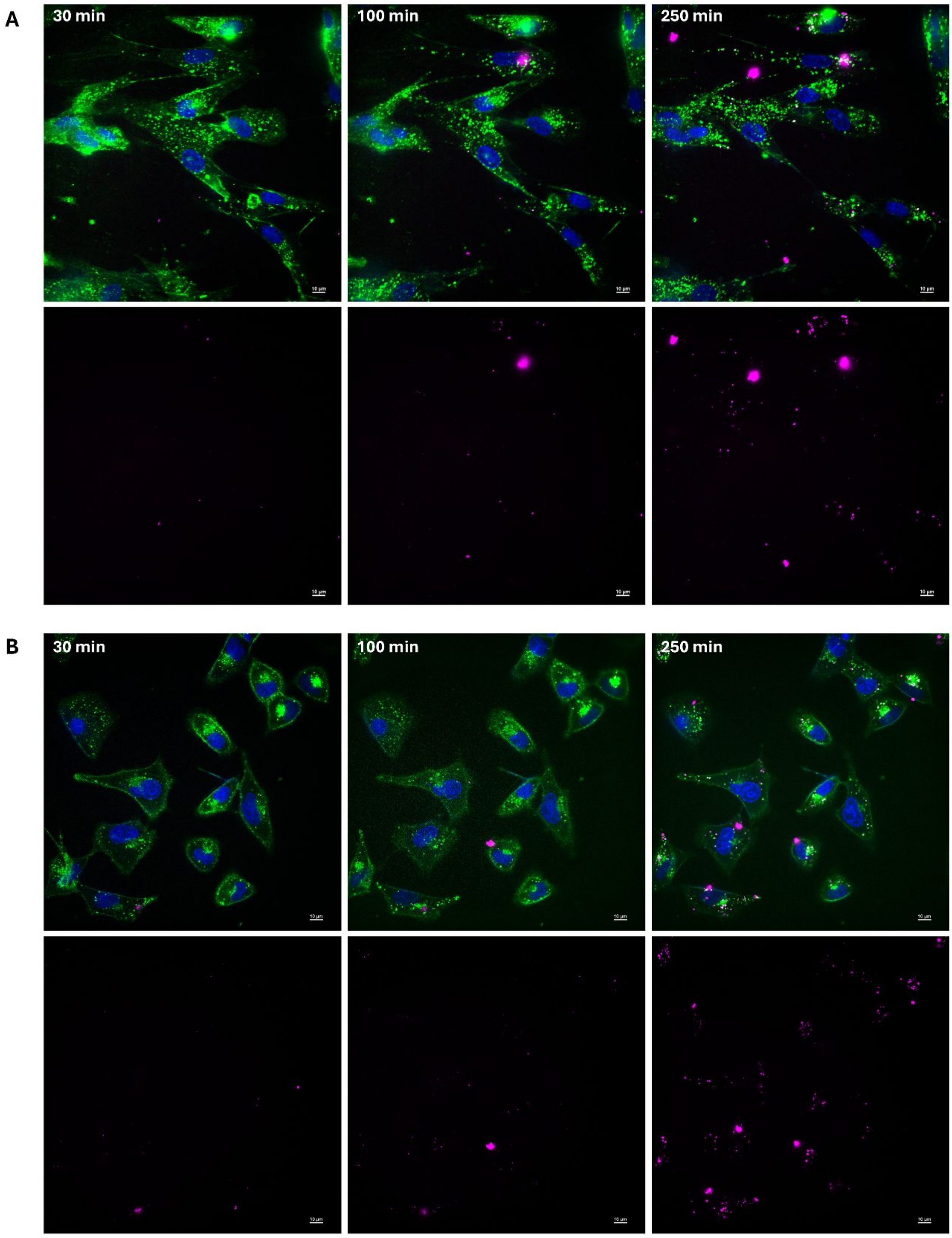

**Figure S4. Images at different time points of fluorescence microscopy time-lapse of CRC-EVs uptaken by lung cells.** Merged and EV channel images from the first (30 min), intermediate (100 min) and last (250 mins) frames of time lapse experiments monitoring CRC-EV internalization in **A)** MRC-5 and **B)** A549 cells. Scale bars: 10μm.

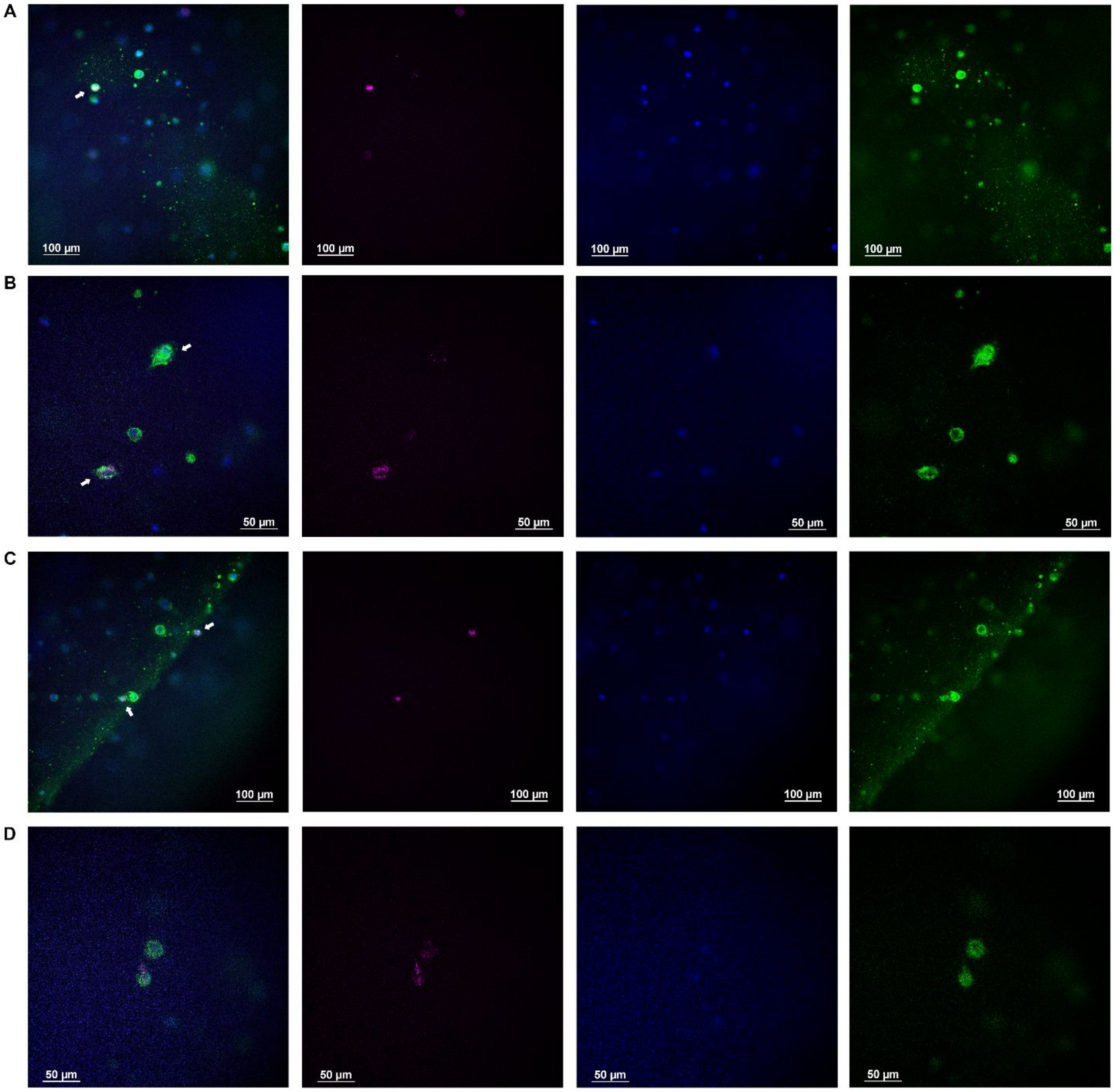


**Figure S5. Fluorescence microscopy images of CRC-EVs uptake by healthy and CRC colon 3D model.** Merged and EV channel images (Cy5-DAPI-FITC) showing CRC-EVs internalization in the

3D model of healthy colon (A-B) and CRC colon (C-D). Magnification: 20x (A and C) and 40 x (B and D).

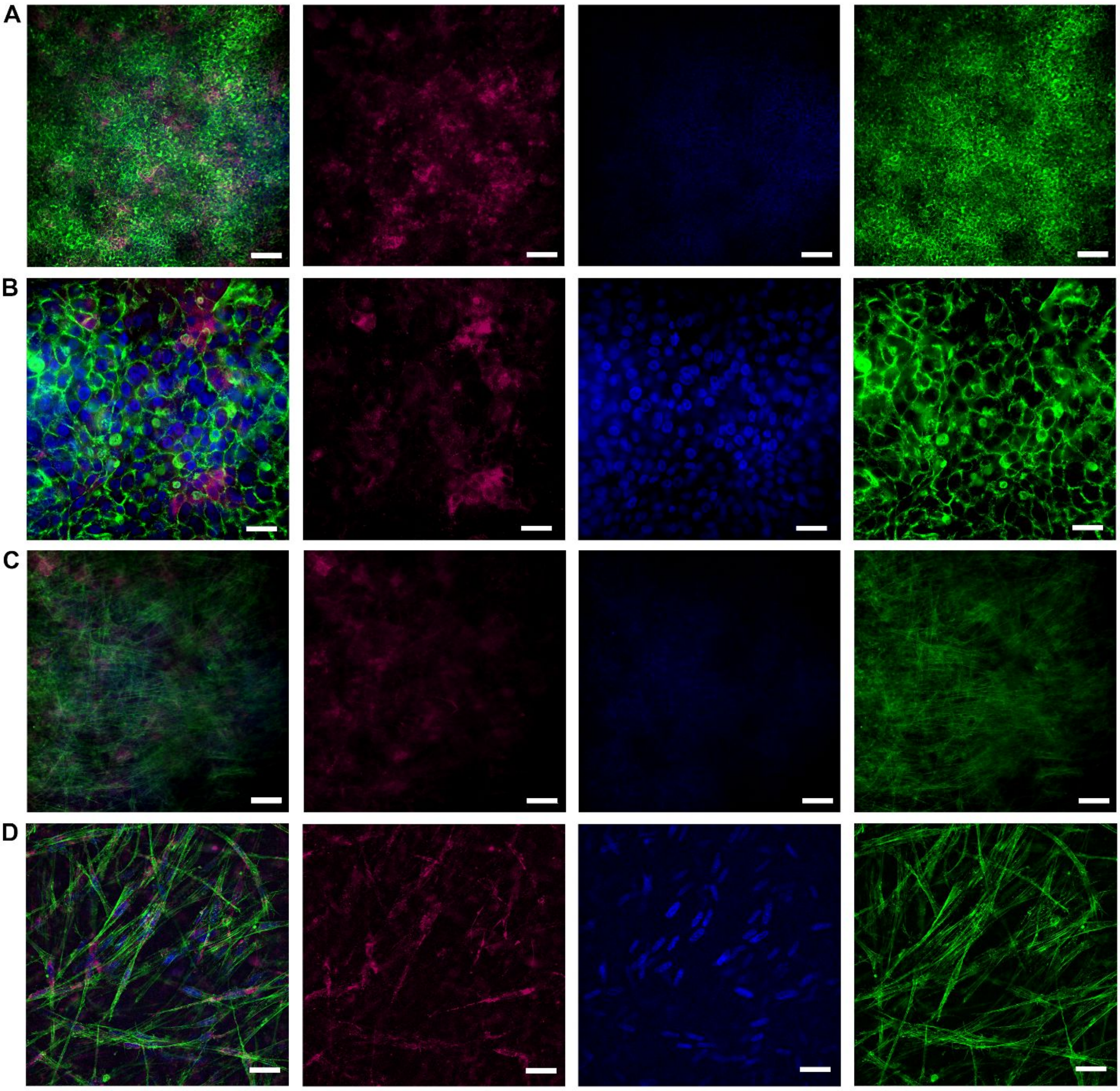


**Figure S6. Fluorescence microscopy images of CRC-EVs uptake by lung 3D model.** Merged and EV channel images (Cy5-DAPI-FITC) showing CRC-EVs internalization in the 3D model of lung by

A549 cells (A-B) and MRC-5 fibroblasts (C-D). Magnification: 20x (A and C, scale bar 100μm) and 60x (B and D, scale bar 20μm).

**Video S1.** Representative video of the co-culture model structure obtained by z-stack acquisition from the epithelium to the stroma after 10 days of culture. A homogeneous distribution of MRC-5 fibroblasts within the hydrogel and a uniform layer of A549 cells was observed.

**Video S2.** Representative video of the co-culture model structure obtained by z-stack acquisition from the epithelium to the stroma after 24 hours of incubation with CRC-EVs. An efficient EV internalization was observed in both A549 cells and MRC-5 fibroblasts.
